# Can Breast Tumors Affect the Oxidative Status of the Surrounding Environment? A Comparative Analysis among Cancerous Breast, Mammary Adjacent Tissue, and Plasma

**DOI:** 10.1155/2016/6429812

**Published:** 2015-11-30

**Authors:** C. Panis, V. J. Victorino, A. C. S. A. Herrera, A. L. Cecchini, A. N. C. Simão, L. Y. Tomita, R. Cecchini

**Affiliations:** ^1^Laboratory of Inflammatory Mediators, State University of West Paraná (UNIOESTE), Campus Francisco Beltrão, 85605-010 Francisco Beltrão, PR, Brazil; ^2^School of Medicine, State University of Sao Paulo (USP), 07112-000 São Paulo, SP, Brazil; ^3^School of Medicine, Pontifícia Universidade Católica (PUC), Campus Londrina, 86051-990 Londrina, PR, Brazil; ^4^Laboratory of Physiopathology and Free Radicals, Department of General Pathology, State University of Londrina (UEL), 86051-990 Londrina, PR, Brazil; ^5^University Hospital, Department of Pharmacy, State University of Londrina (UEL), 86051-990 Londrina, PR, Brazil; ^6^Department of Preventive Medicine, Federal University of São Paulo (UNIFESP), 07112-000 São Paulo, SP, Brazil

## Abstract

In this paper, we investigated the oxidative profile of breast tumors in comparison with their normal adjacent breast tissue. Our study indicates that breast tumors present enhanced oxidative/nitrosative stress, with concomitant augmented antioxidant capacity when compared to the adjacent normal breast. These data indicate that breast cancers may be responsible for the induction of a prooxidant environment in the mammary gland, in association with enhanced TNF-*α* and nitric oxide.

## 1. Introduction

Redox imbalance is a process reported in most of the chronic diseases [1]. The constant activity of reactive species (RS) on lipids, DNA, and proteins promotes critical modifications in cell physiology [2], which can interfere with its normal functioning under chronic inflammatory conditions, such as cancer [3].

Cancer cells constantly experience moderate to high levels of oxidative stress, but curiously moderate to high oxidative stress does not cause immediate cell death [4]. This fact indicates that cancer cells are able to overcome and adapt against redox changes. The sustained oxidative stress promoted during chronic inflammation supports pivotal events to cancer survival, including most of the hallmarks of cancer. This fact has been associated with aberrant activation of transcription factors, induction of protooncogenes, and cumulative acquisition of mutations, which perpetuates the genomic instability of cancer cells [5].

Tumors with enhanced proliferative capacity, as breast cancer, produce high levels of RS during their chronic cycles of ischemia, reperfusion, and angiogenesis, resulting in exceeding growth signaling [6]. It is reported that DNA obtained from breast carcinomas presents greater oxidative damage than the adjacent nontumoral breast [7], suggesting that the tumor cells are more exposed to* in situ* oxidative stress than the proximal or distant nontumoral tissues. On the other hand, this fact suggests that cancer can potentially induce oxidative damage in surrounding normal cells.

Therefore, nontransformed epithelial cells located adjacently to the tumoral tissue may experience variable concentrations of RS generated by the constitutive activation of mitogenic pathways arising from surrounding tumor cells [8]; however, the impact of this event on the homeostasis of nontumoral adjacent cells is unclear. What is known so far is that tumors are “oxidatively stressed” and that in some extension it could be related with the systemic redox changes reported in patients bearing breast tumors [9–11].

Although growing evidence highlights the occurrence of persistent oxidative stress in breast tumors, most of studies have not focused on reporting the redox modifications of breast cancer regarding its healthy counterpart tissue and whether there is a relationship between the tumor oxidative status and the systemic redox profiling. In this context, we proposed to map the oxidative and inflammatory profiles of fresh nonfixed breast tumors and their paired adjacent mammary nontumoral tissue, as well as their respective plasma. To reach these goals, we designed our analysis employing high-sensitivity oxidative stress approaches to investigate the functional redox changes that occurred in tumor microenvironment and its correlation with the circulating levels of proinflammatory/oxidative mediators.

## 2. Material and Methods

### 2.1. Study Design

A series of 321 women with breast cancer were screened from March 2011 to December 2012 at Londrina Cancer Institute, Londrina, Paraná, Brazil. A total of 50 women were included based on the inclusion and exclusion criteria. Inclusion criteria embraced women bearing unilateral tumor with histopathological diagnosis of primary ductal infiltrative carcinoma of the breast, before starting the chemotherapeutic regimen. Exclusion criteria included current smoking, hepatic, cardiac, or renal dysfunction, obesity, use of drugs, hypertension, autoimmune disorders, and diabetes, among other chronic conditions.

Adjacent mammary tissue and tumoral tissue were surgically resected at the moment of tumor withdrawal according to standard procedures, before chemotherapy starting. The adjacent tissue was collected from the most distant point in relation to the tumoral tissue (3 to 4 cm of distance from the macroscopic tumor). Adjacent breast was confirmed as nontumoral by conventional histopathological analysis. Heparinized blood was further collected for analysis. Samples were kept frozen at −86°C until analysis, by at most 2 weeks.

All recommendations of the Reporting Recommendations for Tumor Marker Prognostic Studies (REMARK) criteria [12] were followed throughout this study regarding patient selection, assays performance, and data analysis. Institutional board previously approved all practice and all participants signed informed consent terms. This study is in accordance with the ethical principles for medical research involving human subjects from the Declaration of Helsinki.

Clinicopathological data of cancer patients was collected from medical records and included age at diagnosis, TNM staging, tumor histological type, histological tumor grade, lymph nodal status, tumor size, and presence of distant metastasis.

### 2.2. Immunohistochemical Labeling for Nitrotyrosine (NT)

Nitrotyrosine (NT) is a residue formed by the action of peroxynitrite (derived from the reaction of nitric oxide and superoxide anion) on proteins. Paraffin-embedded sections were heated (30 min, 65°C), deparaffinized, and rehydrated. Sections were treated at room temperature with 2% bovine serum albumin and incubated overnight at 4°C with primary mouse anti-human antibodies against NT-labeled residues (diluted 1 : 300, Santa Cruz Biotechnology, clone sc-32757, USA), previously validated for human samples [13]. The secondary antibody, horseradish peroxidase, and 3,3′-diaminobenzidine (DAB) were provided by the commercial kit (Dako LSAB, Germany). In the last step, sections were weakly counterstained with Harry's hematoxylin (Merck). For each case, negative controls were performed on serial sections by omitting the primary antibody incubation step. The intensity and localization of the immunoreactivity were examined with a photomicroscope (Leica DM 2500 and Leica DFC280, Leica, Germany).

### 2.3. Sample Processing for Determining the Tissue Oxidative Status

Frozen tissue samples were thawed, precisely weighted, and homogenized in sterile saline phosphate buffer 10 mM pH 7.4, at a final concentration of 100 mg tissue/mL. The mixture was centrifuged at 5000 ×g, 4°C during 10 minutes. Supernatants were collected and kept in ice bath until analysis. All described methods used this concentration of tissue homogenate, except when some specific dilution is highlighted. Heparinized blood samples were centrifuged at 1500 ×g, 4°C during 5 minutes. Plasma samples were separated for further analysis. All measurements were conducted at the same day of sample processing.

### 2.4. Measurement of Tissue Lipoperoxidation by High-Sensitivity Chemiluminescence

Lipoperoxidation of adjacent and tumoral tissue was evaluated as previously published, with some adaptations [14]. Aliquots of 250 *μ*L of tissue homogenate (100 mg/mL) were added to 750 *μ*L of saline phosphate buffer 10 mM pH 7.4, with addition of 10 *μ*L of t-butyl 3 mM solution. For plasma analysis, 200 *μ*L of sample was mixed with 780 *μ*L of saline phosphate buffer 10 mM pH 7.4, with addition of 20 *μ*L of t-butyl 3 mM solution. Readings were carried out in a Glomax luminometer (Glomax, Promega). The results were expressed as relative light units (RLU) and the entire curve profile was used as indicator of lipoperoxidation. The area under the curve (AUC) was obtained by area integration.

### 2.5. Determination of Lipid Hydroperoxide Level by the Ferrous Oxidation-Xylenol Orange (FOX) Method

Lipid hydroperoxide concentrations in samples were estimated by the FOX method as published by Victorino et al. [15]. Plasma or tissue homogenate aliquots of 50 *μ*L were mixed with 50 *μ*L of FOX reagent. Samples were incubated for 30 minutes in the dark at room temperature and the absorbance of the supernatant was measured at 550 nm and the results were expressed as *μ*M hydroperoxide/mg tissue.

### 2.6. Carbonyl Content and Antimyeloperoxidase Determination

Carbonyl content was measured as estimate of oxidative injury in proteins [16]. Tissue homogenate or plasma aliquots of 200 *μ*L were added in 2 tubes. Test tubes received 1 mL of dinitrophenylhydrazine (DNPH) 10 mM and blank tubes received 1 mL of HCL 2.5 M. Tubes were incubated during 1 hour in ice bath. After that, samples were successively incubated with 1.25 mL of trichloric acetic acid 20% and 10% in ice bath during 20 minutes each, with centrifugation between incubations (1400 ×g/15 minutes). Supernatants were discarded and pellets were twice treated with 1 mL of an ethanol/water solution (1 : 1). The final precipitates were dissolved in 1 mL of guanidine 6 M and were left for 24 hours at 37°C. Carbonyl content was calculated by obtaining the spectra at 355–390 nm of DNPH-treated samples, employing one blank tube for each test. The obtained peaks were employed to calculate carbonyl concentration using a molar extinction coefficient of 22 M^−1^cm^−1^. Results were expressed as nmol/mL/mg tissue. Myeloperoxidase was detected by a commercial ELISA kit following manufacturer's instructions (IBL International, Germany), and the data were expressed as U anti-MPO/mL.

### 2.7. Total Radical-Trapping Antioxidant Parameter (TRAP)

TRAP was measured in pure breast tissue homogenates (100 mg/mL) or plasma samples (diluted 1 : 50). For TRAP calculation, the induction time of the sample (time for which the sample antioxidants can inhibit the ABAP action) was compared to that of the standard antioxidant (trolox) and expressed as *μ*M trolox/g tissue [17].

### 2.8. Evaluation of Nitrite as Estimate of Nitric Oxide (NO) Levels

NO was estimated by measuring nitrite as previously described [18]. Homogenate or plasma samples (60 *μ*L) were deproteinized, and the supernatants were recovered and incubated with cadmium granules. After 10 minutes, the Griess reagent (Sigma) was added to 200 *μ*L of the supernatants, and the reactions were incubated for 10 minutes at room temperature. The absorbance was read at 550 nm using a standard microplate reader (Multiskan EX, LabSystems, Minnesota, USA). The final results were expressed as *μ*M nitrite/mg tissue.

### 2.9. Estimation of MDA Levels by High-Performance Liquid Chromatography (HPLC)

MDA determinations were made using equipment HPLC-20AT Shimadzu equipped with a LC20AT pump and SPDM20A UV, diode array absorbance detector employing a C18 reverse phase column, as described by [15]. Aliquots of 160 *μ*L of plasma samples, tissue homogenate, or standard solution reacted with 100 *μ*L of 0.5 M perchloric acid. Samples were centrifuged for 5 minutes, 5000 ×g at 4°C. About 180 *μ*L of supernatant was recovered to react with 100 *μ*L of thiobarbituric acid for 30 minutes, at 95°C, and transferred to ice bath to stop reaction. 100 *μ*L of 1 M NaH2PO4, pH 7.0, was added to stabilize sample pH. Further, samples were centrifuged for 10 minutes, 5000 ×g at 4°C. Mobile phase was composed of 65% 50 mM KH2PO4 buffer, pH 7.0, and 35% methanol HPLC grade. Readings were executed at 535 nm during 12 minutes with isocratic flow of 0.8 mL/minute and results were expressed as MDA peak height.

### 2.10. Homocysteine and TNF-*α* Levels

Homocysteine (Axis-Shield Diagnostics, Abbott Diagnostics Division, UK) and TNF-*α* levels (e-Bioscience, USA) were determined in aliquots of 200 *μ*L of plasma or tissue homogenates by using commercial kits. Homocysteine was expressed as *μ*mol/L and TNF-*α* as pg/mL.

### 2.11. Statistical Analysis

All analyses were conducted in triplicate sets. Statistical analysis was performed using GraphPad Prism 5.0, Microsoft Office Excel 2007, and OriginLab 7.5 software. Results were expressed as arithmetic means and errors of the means. Differences among groups were assessed by two-way analysis of variance (ANOVA) with* post hoc* Bonferroni's test for the lipid peroxidation curves and by Student's paired *t*-test for the other parametric parameters. Nonparametric data was analyzed by Mann-Whitney or Wilcoxon matched-pairs tests. Correlations among parameters in plasma and tumoral tissue were also performed using Pearson or Spearman tests. All data were checked using the Grubbs test (GraphPad Quickcalcs) to eliminate significant outliers (*p* < 0.05). *p* < 0.05 was considered statistically significant.

## 3. Results

Table 1 shows the clinicopathological characterization of the 50 patients enrolled in this study. The mean age at diagnosis was 53.8 years, ranging from 31 to 77 years. Most of tumors presented histological grade 2 and the mean tumor size was 2.9 cm. Regarding tumor subtype, a prevalence of luminal tumors was found (30% of patients presented luminal A tumors, 20% were triple negative, 26.6% had HER2 enriched, and 23.4% were typed as luminal B). Most women presented local or locoregional disease (TNM I/II, 83.3%; TNM III, 16.7%), without any presence of distant metastasis. None of the included women were overweight/obese at diagnosis. The number of patients did not allow dividing the groups regarding the molecular subtype.

**Figure 1:**
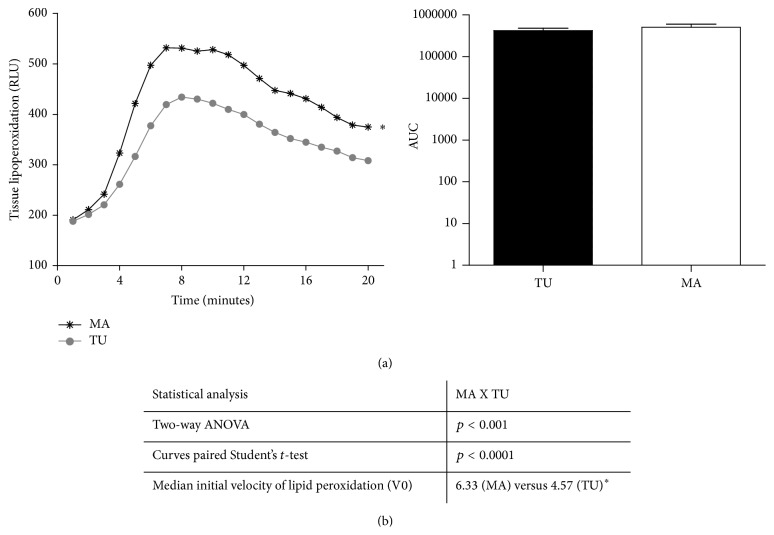
Lipid peroxidation profile of mammary adjacent tissue (MA) and tumoral tissue (TU).

**Figure 2:**
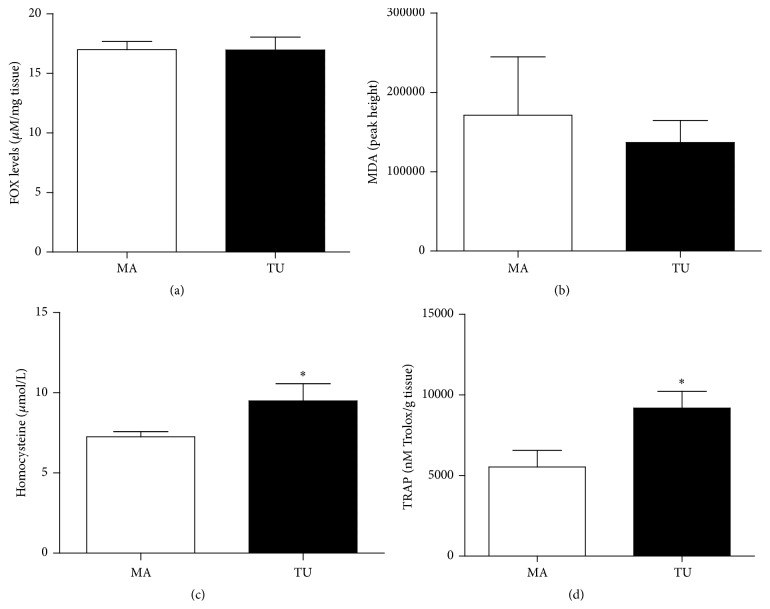
Oxidative stress markers in the mammary adjacent tissue (MA) and the tumoral tissue (TU).

Aiming at characterizing the redox status of adjacent and tumoral breast tissue samples, we performed the analysis of some markers of oxidative damage in lipids and proteins.  Figure 1 shows the lipid peroxidation profile determined by high-sensitivity chemiluminescence. This method allows identifying the oxidative damage of RS on lipidic components located at the plasmatic membrane. As shown, the adjacent mammary tissue presented higher lipid peroxidation status than the tumoral tissue (*p* < 0.0001). Other lipid peroxidation-derived metabolites did not vary (FOX and MDA levels, Figures 2(a) and 2(b)).

Elevated homocysteine levels (from 7.26 ± 0.31 *μ*M/100 mg tissue in adjacent breast to 9.49 ± 1.08 *μ*M/100 mg tissue in tumoral tissue, *p* = 0.0221, Figure 2(c)) were found in the tumoral tissue when compared to the adjacent mammary breast. Antioxidant capacity of tumoral tissue was significantly higher than the adjacent breast (5532 ± 1041 nM trolox/g tissue in adjacent breast and 9181 ± 1041 nM trolox/g tissue in tumor, *p* = 0.0068, Figure 2(d)).

**Figure 3:**
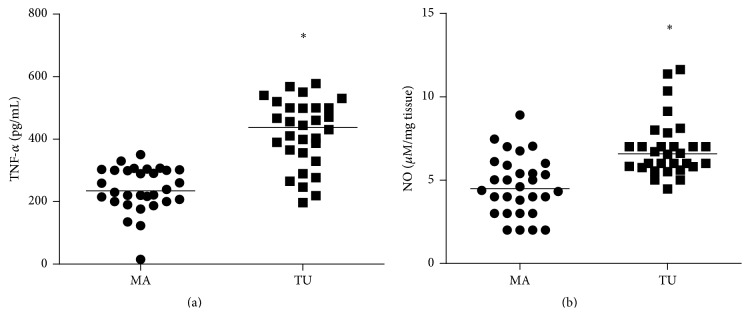
Tumor necrosis factor-alpha (TNF-*α*) and nitrite as estimate of nitric oxide levels (NO) from mammary adjacent tissue (MA) and tumoral tissue (TU).

Tumor samples displayed increased TNF-*α* levels (239.8 ± 13.07 pg/mL of homogenate in adjacent breast and 418.1 ± 19.6 pg/mL of homogenate in tumoral tissue, *p* < 0.001, Figure 3(a)) and NO (4.64 ± 0.32 *μ*M/mg tissue in adjacent breast and 6.89 ± 0.32 *μ*M/mg tissue in tumoral tissue, *p* < 0.001, Figure 3(b)).

**Figure 4:**
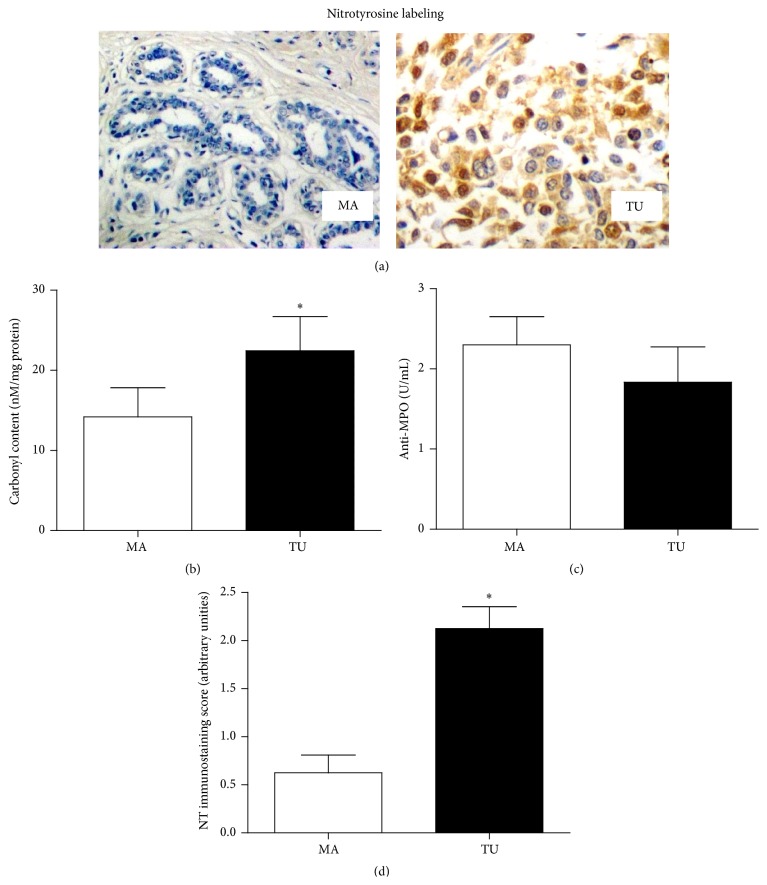
Protein-induced oxidative modifications and MPO detection in mammary adjacent tissue (MA) and tumoral tissue (TU).

The protein-induced oxidative modifications are represented in Figure 4. All tumor samples presented moderate/intense labeling for nitrotyrosine labeling, suggesting a prooxidant role for NO in breast cancer (Figure 4(a)). High carbonyl content (from 12.33 ± 2.56 nmol/100 mg tissue in adjacent breast to 22.39 ± 3.95 nmol/100 mg tissue in tumoral tissue, *p* = 0.0274, Figure 4(b)) was found in breast tissue. Anti-MPO levels did not vary between groups (2.3 ± 0.35 U/100 mg tissue in adjacent breast and 1.83 ± 0.44 U/100 mg tissue in tumoral tissue, *p* = 0.6129). Semiquantitative analysis of nitrotyrosine (Figure 4(d)) showed augmented levels in TU samples when compared to the adjacent normal breast (0.625 ± 0.18 arbitrary unities in MA samples and 2.125 ± 0.226 arbitrary unities in TU samples, *p* = 0.0025).

We further performed Spearman analysis to investigate whether there was some correlation between the oxidative status of tumors and its respective plasma obtained from the same patient (Table 2). All parameters were compared. Significant positive correlations were found with plasmatic versus tumoral TNF-*α* (*p* < 0.001), tumoral nitrotyrosine versus plasmatic NO (*p* = 0.0456), and plasmatic versus tumoral carbonyl contents (*p* = 0.0302).

## 4. Discussion

It is known that oxidative stress is active during the carcinogenic process and correlates with disease prognosis in breast cancer patients [19, 20]. In spite of that, this is the first characterization of the oxidative status of human tumor samples in comparison with matched nontumoral adjacent mammary tissue. Our findings indicate that the breast tumor presents a variable oxidative profile. There was reduced oxidative stress as demonstrated by reduced lipid peroxidative reaction, revealed by the decrease of chemiluminescence and MDA levels and increased total antioxidant capacity (TRAP). Contrarily, nitrosative stress increased as nitrotyrosine labeling and NO was shown to be augmented in tumoral tissue with significant correlation between them.

Protein oxidation also was elevated in tumoral tissue possibly as a consequence of nitrosative stress. Our results showing high levels of TNF besides increased levels of NO and nitrotyrosine suggest a possible cross talk between nitrosative stress and inflammatory mediators in tumor tissue. This cross talk is enforced by the significant correlation between TNF-*α*, NO, nitrotyrosine, and the carbonyl content between tumor and its matched plasma sample. The mammary tissue presents a large content of adipocytes, which provides abundant feedstock for the occurrence of lipid peroxidation reactions. Polyunsaturated fatty acids that contain two or more double bonds are more susceptible to peroxidation [21]. Several RS may abstract the first hydrogen atom to produce a lipid peroxyl radical [22]. In this context, we first evaluated the lipid peroxidation chain by using a chemiluminescence-based analysis. Here, the initiation step is characterized by the ascending part of the curve, which is dependent on the antioxidant content of the tissue [23]. The analysis of this initial reaction revealed that the lipids from adjacent nontumoral tissue were more oxidized than the lipidic content of tumor samples. The occurrence of reduced lipid peroxidation in the breast cancer environment has been reported in nipple aspirate fluids, suggesting a role for the downregulation of lipid peroxidation products in carcinogenesis [24]. Low lipid peroxidation activates several redox signaling pathways that recruit antioxidant induction from the organism to the site of increasing RS production and this configures a survival adaptation [25, 26].

In association with the reduced lipid peroxidation profile of tumors, we further found unaltered levels of lipid peroxidation products in tumor samples, as hydroperoxides (FOX) and malondialdehyde (MDA), suggesting that other low molecular weight substances may affect the lipoperoxidative status of cells. These findings indicate that, in some instance, tumors are protected against lipid peroxidation, potentially by accumulating membrane and intracellular antioxidants that neutralize this process. This fact was corroborated by the enhanced antioxidant capacity detected in tumor samples when compared to normal adjacent tissue, which explains the reduced lipid peroxidation chain observed in breast tumors. Therefore, high antioxidant content inside breast tumors may be able to retard the initiation of lipid peroxidation process, which may have a regulatory role in cell adaptation to oxidative changes.

In spite of the enhanced antioxidant capacity of tumors, we found significant augmented protein carbonylation in the tumoral tissue (but not in the adjacent mammary breast). The carbonylation reaction is an irreversible posttranslational modification that occurred in protein structure that results from the reaction between amino acid residues with the low molecular weight aldehydes generated during the lipid peroxidation process [27–30] and it is reported as an oxidative marker locally produced in the cancerous breast [31].

We further detected increased homocysteine levels in breast tumors, suggesting the attack of thiol residues by RS originated in tumor microenvironment. Homocysteine regulates cell cycle, apoptosis, and oxidative stress responses [32]. A direct relationship between high homocysteine and enhanced carbonyl content has been demonstrated [33], indicating that the augmented carbonylation of tumor proteins can be induced by high homocysteine concentration.

In cancer cells, enhanced activity of proinflammatory cytokines leads to RS production [27]. A recent study of nipple aspirate fluids obtained from patients with breast cancer indicated that high levels of cytokines, as TNF-*α*, are concomitant with augmented protein carbonylation [34] and correlated with elevated systemic homocysteine [35], corroborating the present findings in tumor samples. Therefore, enhanced carbonyl content, homocysteine, and TNF-*α* level seem to comprise a network communicating with each other in the breast cancer microenvironment.

The prooxidative status found here in the tumoral tissue was probably sustained by its proinflammatory nature, as shown by TNF-*α* and NO levels in tumor samples. NO is a multifunctional molecule in cancer. Our data indicate a nitrosative function for this molecule inside breast tumor, as nitrotyrosine was found only in the tumor analysis and not detected in the nontumorous counterpart. In addition, the TNF-driven pathway is constitutively activated in breast cancer [8], inducing NO and increasing [36] and modulating oxidative stress in breast cancer cells [37]. Our previous publications have demonstrated that these patients carrying breast tumors present a variety of oxidative plasmatic modifications, and we have questioned if this fact could be related with tumor oxidative status. Therefore, we performed a correlation analysis aiming to understand the putative association between levels of oxidative stress parameters in plasma versus tumoral tissue. We have found significant correlations between plasmatic versus tumoral levels of TNF-*α*, NO/nitrotyrosine, and carbonyl content. Altogether, these data support that the systemic prooxidative status reported for several studies in breast cancer patients may be correlated with tumor-driven inflammation.

A recent study from our group [16] has demonstrated that the presence of the primary tumor mass is determinant for the sustained proinflammatory systemic status found in women with breast cancer, which included high NO, enhanced oxidative stress, and augmented TNF-*α*. Therefore, in spite of the reduced size of the tumor mass, it seems that the breast tumor microenvironment (cancer cells, infiltrated macrophages, and endothelial cells) is endowed with an enormous capability to promote profound modifications in the host organism, resulting in the persistent inflammatory status provoked here by TNF-*α*, NO, and RS production. This fact cannot be satisfactorily explained by the immune response against the tumor, since women presenting local breast disease have systemically established a Th2 immune status [11].

Another contributing factor to this redox scenario may be the components of tumor stroma. Tumor-associated fibroblasts can affect metabolically their adjacent cells, and cancer cells use the oxidative environment as advantage to obtain nutrients from the surrounding environment [38]. Further, sustained oxidative stress allows cancer-associated fibroblasts to become myofibroblasts, which secrete growth factors and cytokines [39] and yield high ROS generation [40]. This vicious cycle may also affect distant mammary cells by propagating the prooxidant signaling in a paracrine manner, which helps to explain the altered oxidative profile observed here in the distant mammary adjacent tissue.

In conclusion, this set of redox alterations found in breast tumors seems to be necessary to ensure the hallmarks of cancer biology, since RS have been implicated in oncogene activation, genomic instability, chemotherapy resistance, and the metastatic process [5]. Furthermore, it is known that enhanced antioxidant capacity has been strongly associated as an innate tumoral mechanism for acquiring chemoresistance [41]. The presented data point to the existence of a correlation between tumor proinflammatory mediators and their circulating levels, suggesting that the tumor may be a putative source that stimulates the onset of such substances in blood.

## Figures and Tables

**Table 1:** Clinicopathological characterization of patients.

Number of patients	*N* = 50
Mean age at diagnosis (range, years)	53.8 (31–77)
Histological type	
Ductal infiltrative carcinoma	100%
Histological grade	
1 or 2	70%
3	30%
TNM classification	
I/II stage	83.3%
III stage	16.7%
IV stage	None
Tumor size	
Mean (range) cm	2.88 (0.9–5)
Mean BMI (kg/m^2^)	24.2

TNM = tumor-node-metastasis classification, BMI= body mass index.

**Table 2:** Spearman's correlations among the levels of oxidative parameters in plasma and plasma matched tumoral tissue.

Plasma versus tumor	*r* value	*p* value
TNF-*α*	0.8322	*p* < 0.001^*∗*^
NO × nitrotyrosine	0.7771	*p* = 0.0456^*∗*^
Carbonyl content	0.7082	*p* = 0.0302^*∗*^
TRAP	−0.09790	*p* = 0.7621
Homocysteine	0.2562	*p* = 0.5680
MDA	0.5691	*p* = 0.8954
Anti-MPO	0.6598	*p* = 0.6977
Lipid peroxidation	0.5870	*p* = 0.2314
NO	0.5477	*p* = 0.7894

TNF-*α* = tumor necrosis factor-alpha, TRAP = total antioxidant capacity, MDA = malondialdehyde, and MPO = myeloperoxidase.

*∗* indicates significant statistical difference (*p* < 0.05).
